# Sarcopenia in cirrhosis: from pathogenesis to interventions

**DOI:** 10.1007/s00535-019-01605-6

**Published:** 2019-08-07

**Authors:** Maryam Ebadi, Rahima A. Bhanji, Vera C. Mazurak, Aldo J. Montano-Loza

**Affiliations:** 1 Division of Gastroenterology and Liver Unit, Zeidler Ledcor Centre, University of Alberta, 8540 112 Street NW, Edmonton, AB T6G 2X8 Canada; 2 Division of Human Nutrition, University of Alberta, Edmonton, AB Canada

**Keywords:** Muscle loss, Pathways, Mechanisms, Interventions

## Abstract

Sarcopenia (severe muscle depletion) is a prevalent muscle abnormality in patients with cirrhosis that confers poor prognosis both pre- and post-liver transplantation. The pathogenesis of sarcopenia is multifactorial and results from an imbalance between protein synthesis and breakdown. Nutritional, metabolic, and biochemical abnormalities seen in chronic liver disease alter whole body protein homeostasis. Hyperammonemia, increased autophagy, proteasomal activity, lower protein synthesis, and impaired mitochondrial function play an important role in muscle depletion in cirrhosis. Factors including cellular energy status, availability of metabolic substrates (e.g., branched-chain amino acids), alterations in the endocrine system (insulin resistance, circulating levels of insulin, insulin-like growth factor-1, corticosteroids, and testosterone), cytokines, myostatin, and exercise are involved in regulating muscle mass. A favored atrophy of type II fast-twitch glycolytic fibers seems to occur in patients with cirrhosis and sarcopenia. Identification of muscle biological abnormalities and underlying mechanisms is required to plan clinical trials to reverse sarcopenia through modulation of specific mechanisms. Accordingly, a combination of nutritional, physical, and pharmacological interventions might be necessary to reverse sarcopenia in cirrhosis. Moderate exercise should be combined with appropriate energy and protein intake, in accordance with clinical guidelines. Interventions with branched chain amino acids, testosterone, carnitine, or ammonia-lowering therapies should be considered individually. Various factors such as dose, type, duration of supplementations, etiology of cirrhosis, amount of dietary protein intake, and compliance with supplementation and exercise should be the focus of future large randomized controlled trials investigating both prevention and treatment of sarcopenia in this patient population.

## Introduction

Sarcopenia (severe muscle depletion) is a prevalent muscle abnormality in patients with cirrhosis that is related to increased complications both pre- and post-liver transplantation (LT). The pathogenesis of sarcopenia is multifactorial and results from an imbalance between protein synthesis and breakdown. LT evaluation at most centers is comprised of computed tomography (CT) and magnetic resonance imaging (MRI) for evaluation of the vascular and biliary anatomy and as screening for hepatocellular carcinoma (HCC). Recently, secondary analysis of CT images for the evaluation of body composition has been developed as an objective, precise, and gold standard approach to diagnose skeletal muscle abnormalities. CT-measured muscle cross-sectional area (cm^2^) at the level of the third lumbar vertebrate (L3), normalized to the patient’s height and reported as skeletal muscle index (SMI) is a robust indicator of whole-body muscle mass [1]. Skeletal muscle can be quantified on a single slice CT using pre-established radiation attenuation ranges of − 29 to 150 Hounsfield unit (HU) [2]. Choice of software program to quantify muscle mass [3] and use of non-contrast *versus* contrast-enhanced CT scans [4] do not affect the identification of sarcopenia. However, psoas muscle index confers poor performance in identifying sarcopenic patients with higher waitlist mortality risk in cirrhosis demonstrating the need for sensitive measurements of skeletal muscle abnormalities to define mortality-associated cutpoints in these populations [5]. Recently, a specific cutpoint for mortality-associated SMI has been developed in patients listed for LT as SMI < 50 cm^2^/m^2^ in males and < 39 cm^2^/m^2^ in female patients at North American LT centers [6].

The prevalence of sarcopenia in patients with cirrhosis varies within the literature, ranging from 40 to 70% [7]. This discrepancy might be related to divergent quantitative measurements of muscle mass, criteria, and cut-points for sarcopenia, outcomes of interest and study population. Applicability of body mass index (BMI)-dependent cut-points derived from cancer populations is debatable in patients with cirrhosis due to fluid retention. Moreover, divergent outcomes such as overall mortality in patients evaluated for LT, mortality on waitlist in patients listed for LT, post-LT mortality, and short- *versus* long-term outcomes limit the ability to compare the studies.

While extensive data support the prognostic significance of sarcopenia in patients with cirrhosis [8], there are limited data on the pathogenic mechanism of sarcopenia in this population. The aim of this literature review was to summarize the existing knowledge on sarcopenia in patients with cirrhosis, focusing on impact on clinical outcomes, underlying molecular mechanisms, and interventions targeting these specific mechanisms to reverse sarcopenia.

## Clinical importance of sarcopenia in cirrhosis

Sarcopenia, independent of liver function, is an important predictor of mortality pre- [9–11] and post-LT [12], associated with a higher rate of infection and longer hospital stay [13–15], hepatic encephalopathy [16], poor quality of life [17], and increased healthcare cost [18] (Table 1). Similarly, sarcopenia is associated with poor survival in patients with hepatocellular carcinoma (HCC) [19].

**Table 1:** Summary of main studies investigating the association between pre-liver transplantation CT-determined low skeletal muscle index and adverse outcomes in patients with cirrhosis

Author/year	Study population	Sarcopenia definition	Adverse outcome associated with sarcopenia
Pre-liver transplant outcomes			
Carey et al., 2016 [6]	396 patients listed for LT	L3 SMI < 39 cm^2^/m^2^ for women and < 50 cm^2^/m^2^ for men	Higher waitlist mortality
Van Vugt et al., 2018 [18]	224 patients listed for LT	L3 SMI < 44.1 cm^2^/m^2^ for men and < 37.9 cm^2^/m^2^ for women	Increased health-related costs in patients waiting for LT
Bhanji et al., 2018 [16]	675 patients evaluated for LT	L3 SMI < 39 cm^2^/m^2^ for women and < 50 cm^2^/m^2^ for men	Higher risk of hepatic encephalopathy
Post-liver transplant outcomes			
DiMartini et al., 2013 [9]	338 LT recipients	L3 SMI ≤ 38.5 cm^2^/m^2^ for women and ≤ 52.4 cm^2^/m^2^ for men	Longer intensive care unit (ICU) stay, total length of stay, and days of intubation Predictor of discharge to medical facility in men
Montano-Loza et al., 2014 [14]	248 LT recipients	L3 SMI ≤ 41 cm^2^/m^2^ for women and ≤ 53 cm^2^/m^2^ for men with body mass index (BMI) ≥ 25 and ≤ 43 cm^2^/m^2^ in patients with BMI < 25	Longer hospital stay and higher incidence of bacterial infections within the first 90 days following LT
Kuo et al., 2019 [116]	126 patients undergoing urgent evaluation and LT	L3 SMI < 48 cm^2^/m^2^ for men	Post-LT mortality in acutely ill men
Bhanji et al., 2019 [117]	293 LT recipients	L3 SMI < 39 cm^2^/m^2^ for women and < 50 cm^2^/m^2^ for men	Progressive worsening of sarcopenia in the interval between LT evaluation and post-LT Increased post-LT length of hospital stay

*L3* third lumbar vertebrate, *LT* liver transplantation, *SMI* skeletal muscle index

Emerging evidence suggests that male sex is a risk factor associated with sarcopenia development [20]. Sarcopenia is more prevalent in male patients with cirrhosis [6, 21] and it is in these patients that the risk of mortality is mainly observed in. In female patients, both sarcopenia [6] and low subcutaneous adipose tissue seem to be associated with mortality [22]. Differences in fat distribution and metabolism [23], skeletal muscle substrate metabolism [24], and hormonal characteristics by sex [25] might partially explain this discrepancy.

Given the poor correlation between muscle mass and liver function, sarcopenia has been considered as an addition to the Model for End-Stage Liver Disease (MELD) score. When sarcopenia exists, it is equivalent to adding 10 extra points to the patients’ MELD score [10]. This score led to an improvement in mortality prediction in comparison to the MELD score, but was mainly in patients with low MELD [10, 26]. Considering the clinical significance of sarcopenia in predicting outcomes in patients with cirrhosis, early identification of muscle biological abnormalities and the underlying mechanisms is a prerequisite for clinical trials assessing interventions based on modulation of specific mechanisms.

## Skeletal muscle characteristics and metabolism

Skeletal muscle makes up 40% of body weight and is mainly involved in mechanical activity requiring muscle fiber contractions. In response to muscular contractions, myocytes synthesize and release proteins called myokines. Myokines not only maintain muscle mass, function, and strength, but also regulate metabolism in muscles and other tissues and organs, including liver and adipose tissue [27]. Myostatin is the first identified myokine and is highly expressed in atrophic skeletal muscles. Activation of myostatin-mediated signaling stimulates protein catabolic processes, prevents protein synthesis and inhibits muscle growth [28]. It also impedes myogenesis by inhibiting activation of satellite cells; these are the muscle-resident myogenic stem cells [27]. Besides, various cytokines and growth factors such as insulin-like growth factor 1 (IGF-1), fibroblast growth factor, interleukin (IL-4), and interleukin 6 (IL-6) regulate skeletal muscle growth and atrophy.

Protein turnover, the balance between protein synthesis and degradation, regulates muscle mass. Several factors such as cellular energy status, availability of metabolic substrates [e.g., branched-chain amino acids (BCAA)], alterations in the endocrine system (insulin resistance, circulating levels of insulin, IGF-1, corticosteroids, testosterone), cytokines, myostatin, and exercise are involved in regulating muscle mass [29]. Skeletal muscle mass is positively regulated through the Akt-mediated mTOR signaling pathway [30]. Activation of Akt in response to insulin or IGF-1, stimulates mammalian target of rapamycin complex 1 (mTORC1) which plays a key role in mediating the effect of mTOR on protein synthesis. Muscle degradation is regulated by three major proteolytic systems including caspase-mediated protein cleavage, ATP-dependent ubiquitin–proteasome systems, and autophagy. Myofibril cleavage by caspase-3 provides substrates for the ubiquitin–proteasome system [29]. The ubiquitin–proteasome system is key in targeting and conjugating proteins with ubiquitin, which is followed by subsequent degradation by the 26S proteasome. Its activation mainly requires glucocorticoids and impaired insulin/IGF-1 signaling [31].

Forkhead box O (FoxO) proteins are in the family of transcription factors that regulates a wide range of atrophy-related genes in muscle, including Fbxo32 (atrogin1), Trim63 (MuRF1), and autophagy genes [32]. Major FoxO family members in skeletal muscle are FoxO1, FoxO3, and FoxO4. The expression of FoxO1 and FoxO3 is up-regulated in catabolic conditions such as cancer cachexia, sepsis and fasting which require nuclear localization to stimulate ubiquitin/proteasome and autophagy/lysosome pathways [33]. Inhibition of Akt increases translocation of FoxO from the cytoplasm to the nucleus and, consequently, induces expression of the main atrophy-related ubiquitin ligases atrogin-1 and MuRF-1, leading to reduction in muscle size and number [34].

Autophagy, programmed cell death, plays an important role in muscle protein degradation. As the myofiber atrophies, several cellular components such as myofibrillar proteins, mitochondria, and nuclei need to be degraded. Autophagy is also prompted in response to cellular stress such as energy exhaustion or nutrient starvation to provide nutrient supply for the cells [29]. During energy deficiency, AMP-activated protein kinase (AMPK) pathway is activated to reduce ATP-consuming processes. AMPK activation not only suppresses mTORC1 [35], but also stimulates myostatin signaling in damaged muscle [36]. Phosphorylation of FoxO3 by AMPK in Akt-independent sites stimulates its transcriptional activity [36] to induce expression of atrogin1 and MuRF1 under these conditions [37].

Pro-inflammatory cytokines are major activators of increased muscle protein degradation. IL-6 has a paradoxical impact on muscle metabolism. Although it stimulates proliferative capacity of stem cells and subsequently activates myogenesis in a transient manner, its long-lasting elevation in chronic diseases contributes to muscle atrophy, in combination with other mediators [38]. Tumor necrosis factor-α (TNF-α) promotes activation of transcription factor nuclear factor κB (NFκB) [29]. NFκB nuclear translocation leads to transcriptional activation of MuRF1, atrogin1, and protein degradation augmentation [39]. Overall, a multifaceted synergy within a complex of cytokines, rather than a single cytokine is the major driver of muscle atrophy in catabolic diseases [40].

## Nutritional and metabolic alterations of sarcopenia in cirrhosis

Chronic catabolic conditions such as cancer cachexia, increased energy expenditure, reduced food intake due to loss of appetite, early satiety, treatment side effects or gastrointestinal motility impairments, alterations in circulating levels of hormones such as insulin, catecholamines are factors contributing to muscle atrophy [41]. In line with other chronic wasting conditions, increased energy expenditure, physical inactivity, low energy intake (< 30 kcal/ ideal body weight) as well as decreased food intake in the setting of ascites and early satiety are factors contributing to depletion of body fat and protein stores in cirrhosis [42–44]. Presence and degree of malnutrition should be identified in all patients with cirrhosis by implementing nutritional screening and assessments. Although subjective global assessment (SGA) is the gold standard method for malnutrition identification, assessment of sarcopenia by CT performs better at predicting adverse clinical outcomes in patients with cirrhosis when compared to the SGA [45].

Metabolic adaptation to starvation is to preserve muscle mass by catabolizing adipose tissue; though, following extended periods of starvation, skeletal muscle protein is also degraded to provide gluconeogenic substrates. Cirrhosis is a condition of accelerated starvation with an impaired adaptive response to fasting, due to impaired hepatic function. Within 10 h of fasting in patients with cirrhosis, fatty acid oxidation, muscle and hepatic glycogen reduction occurs in an equivalent manner to what would be observed in healthy subjects after 3 days of starvation [46]. Hepatic failure and associated nutritional, metabolic, and biochemical deficiencies in chronic liver diseases lead to alterations in whole-body protein homeostasis. Therefore, muscle loss in patients with cirrhosis and accelerated starvation might serve as a compensatory mechanism to provide glucose for the liver.

Hepatic gluconeogenesis and fatty acid oxidation are elevated in cirrhosis due to limited hepatic glycogen content [42]. Lactate and alanine produced by muscle glycogen and protein hydrolysis and glycerol released form adipose tissue are delivered to the liver and served as substrates for gluconeogenesis. Rate of effective hepatic blood flow is the main determinant of uptake of gluconeogenic substrates [47]. Lipolysis of triglyceride in adipose tissue produces non-esterified fatty acids and glycerol, and fatty acid β-oxidation produces ATP required for gluconeogenesis. Although adipose tissue lipolysis is increased in patients with cirrhosis, impaired hepatic uptake of glycerol [47] and minor contribution of glycerol to gluconeogenesis in cirrhosis [48] limit gluconeogenesis from glycerol. It should be noted that nutritional and metabolic alterations alone do not explain low muscle mass in cirrhosis and factors other than nutrient intake and hypermetabolism may contribute to muscle loss in cirrhosis.

## Pathophysiology of sarcopenia in cirrhosis

**Fig. 1:**
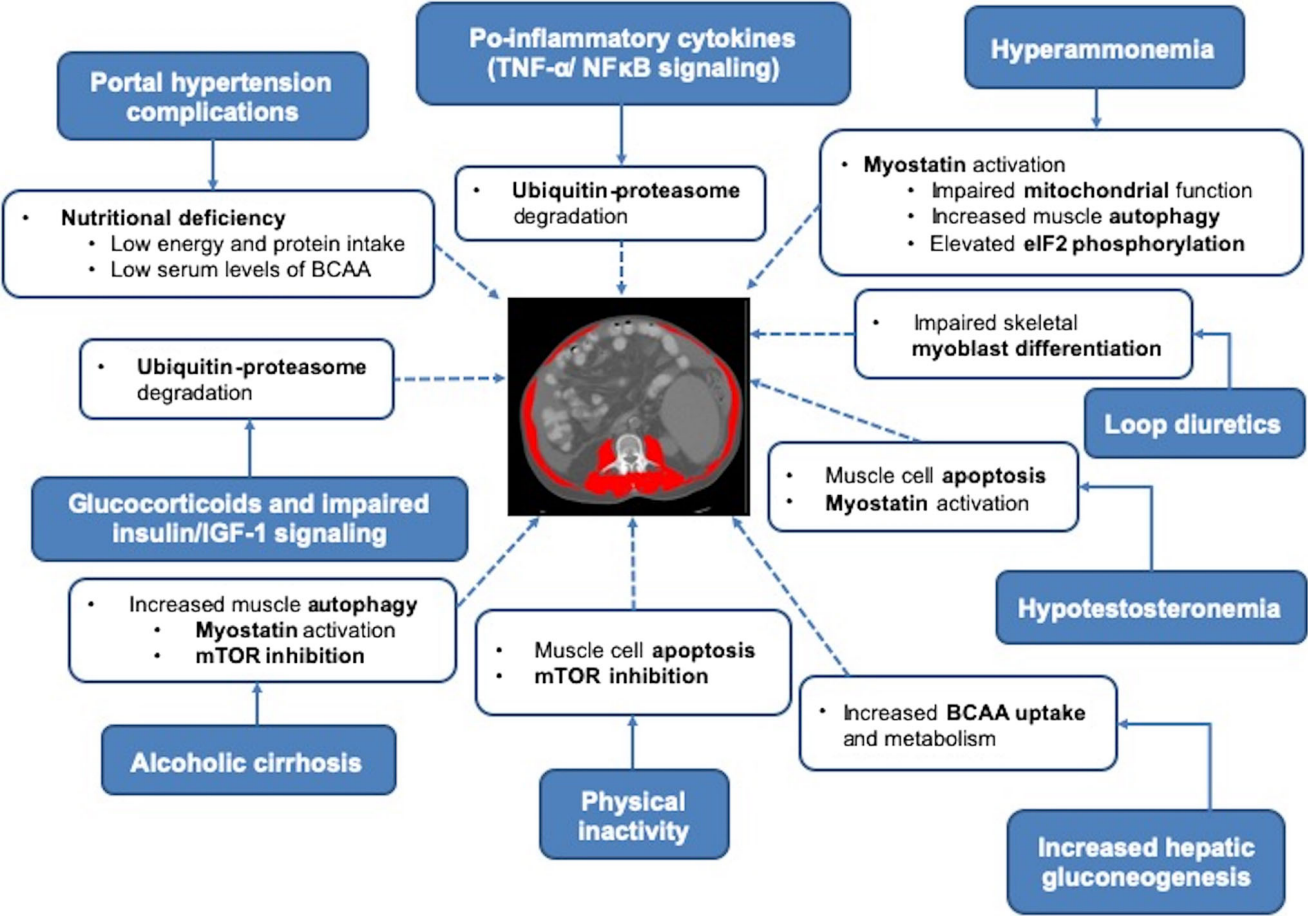
Summary of factors and pathways contributing to sarcopenia in cirrhosis. Numerous factors such as portal hypertension complications, pro-inflammatory cytokines, hyperammonemia, loop diuretics, hypotestosteronemia, physical inactivity, elevated hepatic gluconeogenesis, impaired insulin/IGF-1 signaling and alcoholic cirrhosis associate with sarcopenia in cirrhosis. Several signaling pathways including ubiquitin–proteasome degradation, myostatin activation, impaired mitochondrial function, NFκB signaling, mTOR inhibition, apoptosis and elevated eIF2 phosphorylation are involved in sarcopenia in cirrhosis. *BCAA* branched-chain amino acids, *eIF2* eukaryotic initiation factor 2, *IGF-1* insulin-like growth factor 1, *NFκB* nuclear factor κB

Understanding muscle biological abnormalities and underlying mechanisms is necessary to facilitate the development of interventions to improve muscle mass in cirrhosis. The exact mechanism contributing to muscle atrophy in cirrhosis has not been clearly identified. Hyperammonia, muscle autophagy, lower levels of testosterone, growth hormones or BCAA, are considered to be potential contributors to sarcopenia (Fig. 1). However, most of the research has been done on animal models and the studies of putative mechanisms of sarcopenia in cirrhosis are scare in clinical populations.

### Experimental studies

Hyperammonemia is commonly seen in cirrhosis and plays a major role in the pathogenesis of hepatic encephalopathy (HE). Sarcopenia has been identified as the main risk factor for HE development [16]. Hepatic dysfunction and portosystemic shunting limit the liver’s ability to detoxify ammonia in cirrhosis; therefore, skeletal muscle plays a compensatory role in ammonia metabolism and clearance [49]. Ammonia detoxification in muscle occurs in mitochondria [50] and requires glutamine formation; this is dependent on glutamate production from α-ketoglutarate. Depletion of glutamate in hyperammonemic states requires catabolism of BCAA to provide carbon skeletons for the tricarboxylic acid (TCA) cycle, re-forming α-ketoglutarate and maintaining sufficient levels of glutamate for ammonia detoxification and subsequently for glutamine formation. Glutamine is released into the circulation in exchange with BCAA [51]. Increased BCAA uptake and metabolism by muscle in hyperammonemic states leads to depletion of BCAA required for protein synthesis [52].

Hyperammonemia decreases muscle protein synthesis by (1) myostatin activation via NF-κB-dependent pathways [53] (2) promoting oxidative stress and impaired mitochondrial function due to the loss of the TCA cycle intermediate, α-ketoglutarate [50]; and (3) increased skeletal muscle autophagy of proteins nitrated due to hyperammonemia [53]. As a result, hyperammonemia in skeletal muscle collectively results in mitochondrial dysfunction, increased reactive oxygen species formation and oxidative stress which in turn can cause muscle protein and lipid oxidative damage, further exacerbating sarcopenia [50, 54]. Moreover, increased phosphorylation of the eukaryotic initiation factor 2 (eIF2), an important regulator of protein synthesis involved in translation initiation, impairs protein synthesis in hyperammonemic states [55].

Impact of portosystemic shunting in the etiology of sarcopenia has been studied in male Sprague–Dawley rats who underwent portacaval anastomosis (PCA). Lower muscle mass in PCA rats compared to sham-operated rats was associated with significantly lower testosterone levels, and higher plasma ammonia levels with no changes in plasma IGF-1 levels. During the early weeks following PCA, expression of genes involved in protein breakdown (components of the ubiquitin proteasome pathway) increased. At weeks 3 and 4, post-PCA, lower protein synthesis evidenced by lower skeletal muscle IGF-1 expression resulted in an increased expression of myostatin and lower expression of satellite cell markers. Ex vivo culture of murine myotubes exposed to ammonium acetate revealed hyperammonemia-associated autophagy with no alteration in proteasome activity [56]. Mitochondrial dysfunction and low cellular levels of ATP in a hyperammonemic state impair proteasome activity and may explain the lack of activation of ubiquitin-mediated muscle proteolysis [57]. Compared to pair-fed controls, portacaval shunting led to muscle loss indicating that metabolic changes associated with shunting and hyperammonemia, rather than calorie restriction, may be responsible for muscle depletion [58]. PCA also increased AMPK expression which diminished phosphorylation of muscle mTOR with no changes in Akt phosphorylation [59].

Myostatin may have a significant role in diminished muscle protein synthesis in portosystemic shunting. Administration of follistatin, an antagonist of myostatin, decreased myostatin protein expression, and consequently increased muscle mass and protein synthesis in PCA animals by reversing impaired mTOR phosphorylation and decreasing AMPK expression. However, myostatin activity might be regulated via post-translational modifications, as lower protein but not mRNA expression of myostatin was observed in PCA rats following follistatin treatment [59].

Pro-inflammatory cytokines are thought to be the primary catabolic mediators in skeletal muscle. In cirrhosis, higher levels of systemic inflammatory cytokines including TNF-α, IL-1, and IL-6 have been reported [60]. However, the impact of systemic and local muscle inflammation in sarcopenia development has not been clearly defined in cirrhosis. Levels of TNF-α were higher in the muscle of a rat model with biliary cirrhosis compared to controls and significantly correlated with free and conjugated ubiquitin. Despite no changes in food intake, the activation of ubiquitin–proteasome pathway through TNF-α/ NFκB signaling was the main reason for muscle loss in this animal model of cirrhosis induced by bile duct ligation [61].

Though animal models are required to promote our understanding of cirrhosis-associated muscle loss, they have important limitations. Each model may signify only certain features of human cirrhosis and choice of animal model should be based on research objectives. For example, PCA is the model of portosystemic shunting with complete portal venous deprivation without any basic liver dysfunction and portal hypertension [62]. Skeletal muscle loss in PCA animals is mainly related to the impact of hyperammonia on muscle metabolism. Therefore, the results of these studies examining the mechanisms underlying muscle loss should be interpreted with caution as each specific model, in various stages of growth and treatment can affect muscles in a different manner and not necessarily represent the clinical course of cirrhosis (Fig. 1).

### Clinical studies

Limited number of clinical studies exists regarding the mechanisms underlying muscle atrophy in cirrhosis, and biological differences between animal and humans limit generalizing results between preclinical and clinical studies.

Prevalence of sarcopenia in patients with cirrhosis varies according to the underlying etiologies for cirrhosis. The highest frequency of sarcopenia was observed in patients with alcoholic cirrhosis [9]. Increased muscle autophagy, but not proteasome activity, was the main contributor to sarcopenia in alcoholic cirrhosis [63], as ethanol inhibits ubiquitin proteasome components [64]. Alcohol also inhibits skeletal muscle protein synthesis in experimental studies via myostatin activation or by directly inhibiting mTOR [65] without any infiltration of inflammatory cells [63]. Lastly, progressive muscle loss in patients with alcoholic cirrhosis might be related to the synergistic impact of ethanol and hyperammonemia in these patients [63].

Transjugular intrahepatic portosystemic shunt (TIPS) has been frequently used in patients with portal hypertension to control refractory ascites and variceal bleeding in cirrhosis. In patients who received TIPS, pre-existing sarcopenia was associated with the higher incidence of HE following the procedure. This emphasizes the importance of strategies to preserve muscle mass prior to TIPS to reduce HE occurrence [66]. Elevated muscle mass following TIPS has been associated with lower blood ammonia levels and risk of HE post-TIPS [67]. In addition, it has been associated with decreased mortality [68], independent of liver function. A comprehensive review of the literature shows that sarcopenia reversal following TIPS might be related to reversal of portal hypertension complications and subsequent improvement in nutritional status, lower plasma leptin levels, associated improvement in appetite or reduction in muscle AMPK phosphorylation, lower skeletal muscle myostatin levels, increase in IGF-1 and improved insulin sensitivity [62].

Ubiquitin–proteasome degradation might be involved in muscle loss in patients with cirrhosis. The mRNA expression of rectus abdominis MuRF1, but not myostatin and atrogin1, was significantly higher in malnourished (SGA B/C) patients with cirrhosis compared to well-nourished patients [69]. Although these patients were sarcopenic according to mid-arm muscle circumference measurement, its accuracy is still being debated as a practical prognostic anthropometric measure.

Reduced number of mitochondria or impaired mitochondrial function in skeletal muscle has been observed in patients with cirrhosis Child–Pugh class B and C [70]. Reduced mitochondrial rate of ATP synthesis in skeletal muscle may be associated with the reduction in energy demanding processes such as protein synthesis. Given the major role of mitochondria in regulation of muscle metabolism, future investigations are warranted to elucidate the consequence of interventions in maintaining not only mitochondrial biogenesis, but also function.

Myokines play an important role in regulating metabolism in muscle and other tissues; however, it is not clear whether their production is altered in patients with sarcopenia and cirrhosis. In patients evaluated for LT, there is a significantly higher level of myostatin. However, no correlation has been shown between myostatin levels and calf circumference; this may be related to a small number of patients in this study [71].

Other mechanisms might also be involved in pathogenesis of sarcopenia in cirrhosis. For instance, bacteria and bacterial product transfer into the bloodstream can prompt the release of cytokines implicated in muscle atrophy [72]. Excessive lipid flux into the skeletal muscle and accumulation of lipid-derived intermediates such as diacylglycerol (DAG) and ceramides in skeletal muscle can lead to mitochondrial dysfunction and insulin resistance [73]. To our knowledge, the influence of bacterial translocation and lipid-derived mediators in sarcopenia development in cirrhosis has not been investigated.

Polypharmacy has also been identified as a risk factor for clinically relevant sarcopenia [74]. Loop diuretics are commonly used to treat edema or ascites in patients with cirrhosis. Use of a loop diuretic (> 20 mg) has been linked to accelerated muscle loss and poor survival in patients with cirrhosis [75]. The exact mechanism by which loop diuretics participate in muscle loss in cirrhosis is not clear. However, furosemide and bumetanide, commonly used loop diuretics, impaired skeletal myoblast differentiation in murine skeletal muscle cells ex vivo [76]. Long term administration of other medications such as corticosteroids [77] and statins [78] for co-morbidities in patients with cirrhosis may likewise play a role in muscle depletion.

Sex differences in skeletal muscle metabolism [24] should be considered when investigating the mechanisms underlying muscle loss in cirrhosis. Overall, female muscle has capability to accumulate 57% more lipids than men in non-obese state [79]. Although capacity to produce energy from glucose and ATP seems to be equal in men and women, higher proportion of oxidative fibers and greater cellular availability of fatty acids in women lead to the greater fatty acid utilization during conditions of high ATP demand. Lower glucose oxidation in acute states suggests high metabolic flexibility in women to adjust substrate oxidation in accordance with nutrient availability [24]. In agreement with this notion, we recently demonstrated the importance of subcutaneous adipose tissue in survival of female patients with cirrhosis, while muscle mass was the predictor in male patients [22]. Thus, the metabolic pattern that determines fat loss in female patients with cirrhosis is comparable to states of chronic diseases or starvation while the metabolic pattern of muscle loss in male patients with cirrhosis imitates the critical diseases [80].

Collectively, hyperammonemia, elevated autophagy, proteasomal activity, lower protein synthesis, and impaired mitochondrial function play an important role in muscle depletion in cirrhosis (Fig. 1). Comprehensive elucidation of protein expression and associated pathways that might be altered in patients with sarcopenia and cirrhosis requires further investigation.

## Fiber-type specificity of skeletal muscle atrophy

Besides skeletal muscle mass, muscle fiber size and type might be affected in various physiological and pathological conditions. Skeletal muscle fibers are categorized as slow (Type I) and fast-twitch fibers (Type II). Slow-twitch type I are oxidative fibers of narrow diameters with a large number of mitochondria, whereas IIA are fast oxidative-glycolytic medium size fibers. Type IIB and IIX fibers are fast-twitch glycolytic fibers with wide diameters and few numbers of mitochondria; however, type IIB fibers do not exist in humans [81]. Rates of both muscle synthesis and degradation are higher in muscles comprised of slow-twitch fibers compared to fast muscles [32]. Following exposure to the same stimulus, various skeletal muscles and fiber types respond differently. With regard to the muscle type, lower sensitivity to starvation [82] and catabolic effects of corticosteroids was observed in slow muscles, such as the soleus which is mostly composed of slow-twitch fibers [32], suggesting greater atrophy of type II fast-twitch glycolytic fibers compared to the type I oxidative fibers. Hybrid fiber types are intermediate fibers with transitional phenotypes, connecting the gaps between pure fiber types (shown in bold): I ↔ **I/IIA** ↔ IIA ↔ **IIA/IIX** ↔ IIX ↔ **IIX/IIB** ↔ IIB [83]. Muscle fiber shifts toward fast-twitch fibers (higher expression of IIA/IIX fibers) in cancer patients [84] and following five weeks of bed rest [85], as potential adaptations to muscle disuse. No significant change in fiber-type distribution was observed following bed rest, but the number of fibers expressing mRNA for IIX significantly increased suggesting that morphological changes might occur at later stages compared to changes in corresponding transcripts [85].

Lipids are stored in muscle in the form of intramuscular fat between muscle fibers, intramyocellular lipid droplets within the myocytes and intermuscular adipose tissue found within the fascia surrounding muscle. Lipid accumulation patterns may vary according to the muscle fiber composition. Higher accumulation of intramyocellular lipid droplets was detected in type II fibers than type I fibers in slow-twitch muscles [86]. However, type I fibers accumulate more intramyocellular lipid droplets with aging and obesity [87].

**Fig. 2:**
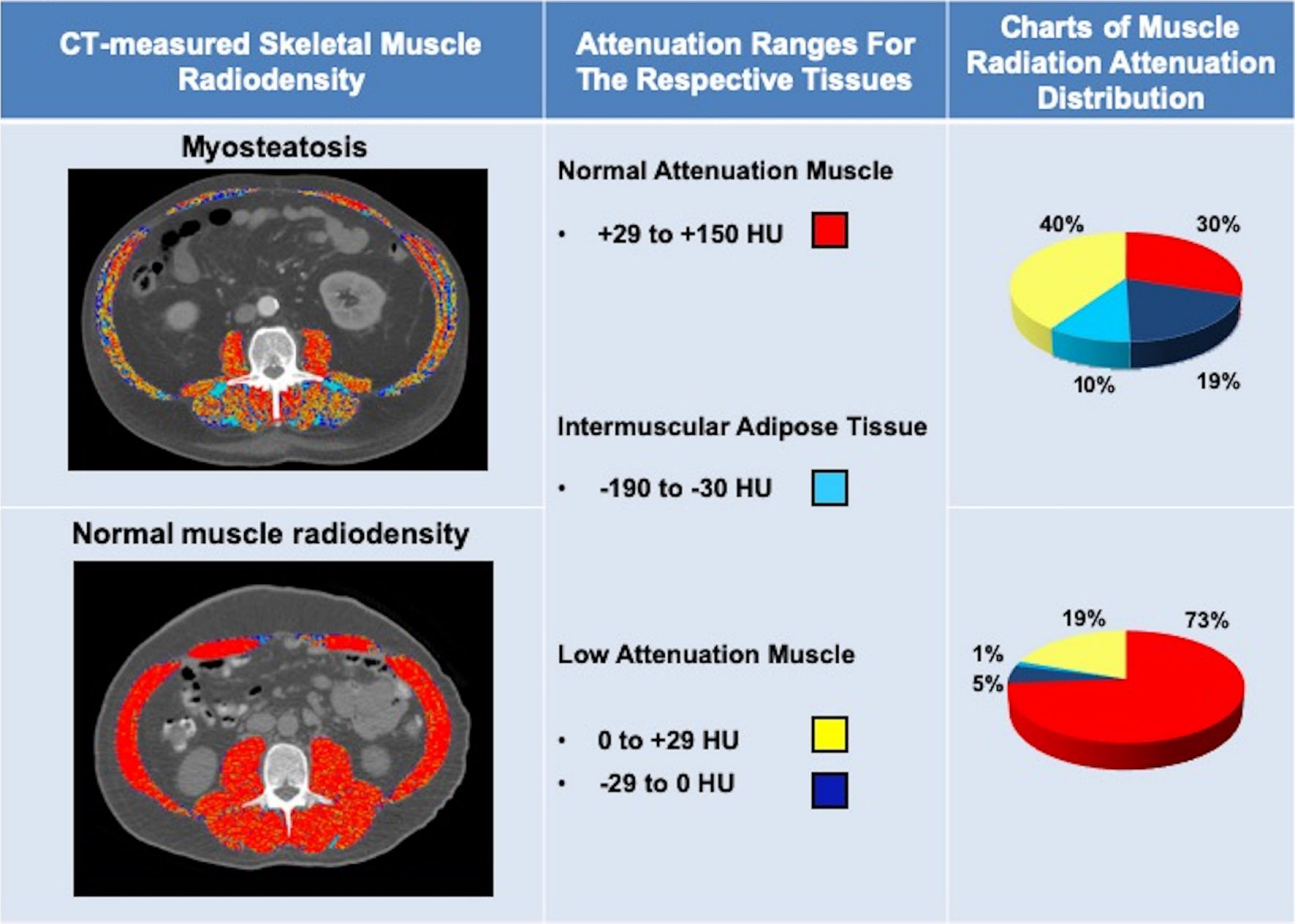
Computed tomography images used for the muscle radiodensity assessment in patients with cirrhosis. Comparison of two patients with cirrhosis and similar BMI. Attenuation ranges used for the analysis of normal attenuation (red), low attenuation region 1 (yellow), and low attenuation region 2 (dark blue) muscle, as well as intermuscular adipose tissue (IMAT; teal), are shown. In patient with low muscle attenuation (34 HU) or myosteatosis, less than half of the muscle cross-sectional area falls within the normal attenuation range whereas in patient with normal muscle attenuation (47 HU, no-myosteatosis), muscle with the normal attenuation range is predominant

Myosteatosis (pathological fat accumulation in skeletal muscle) is a radiologically identified abnormality in skeletal muscle which is defined as attenuated mean skeletal muscle radiodensity (HU) on CT. In Fig. 2, we present the comparison of two patients with cirrhosis and similar BMI, with and without myosteatosis according to the muscle attenuation. Impaired lipid metabolism and oxidation [88], mitochondrial dysfunction, and age-related differentiation of muscle stem cells into adipocytes [89] are potential pathological contributors to myosteatosis. Increased lipid accumulation within muscle fibers may lead to muscle fibers atrophy over time [90]. Although myosteatosis may result in the transformation of the muscle fibers from type II to type I [91], fiber type’s specific lipid accumulation has not been yet defined in cirrhosis.

Fiber-specific skeletal muscle atrophy is mainly related to FoxO family and NF-κB signaling in fast-twitch glycolytic fibers [33]. On the other hand, slow-twitch oxidative fibers are more resistant to atrophy due to high levels of peroxisome proliferator-activated receptor gamma coactivator 1 alpha (PGC-1α) [33, 34]. The PGC-1α is a family of transcriptional coactivators that promotes mitochondrial biogenesis and oxidative metabolism, necessary for maintaining energy homeostasis in muscle. PGC-1α stimulates fiber-type switching from glycolytic toward more oxidative fibers. PGC-1α suppresses protein degradation by inhibiting the transcriptional activity of FoxO3, NFκB, and preventing the expression of critical ubiquitin ligases, atrogin1, and MuRF1 [34]. Exercise induces expression of PGC-1α which protects skeletal muscle from atrophy.

Association between sarcopenia in cirrhosis and muscle fiber type atrophy has not been clearly demonstrated. Only one study found that an association between hyperammonemia and enhanced skeletal muscle BCAA catabolism and oxidation is predominant in muscle with high amount of fast-twitch fibers [51]. Thus, similar to other chronic diseases such as cancer cachexia, atrophy of fast-twitch fibers is more likely to occur.

**Fig. 3:**
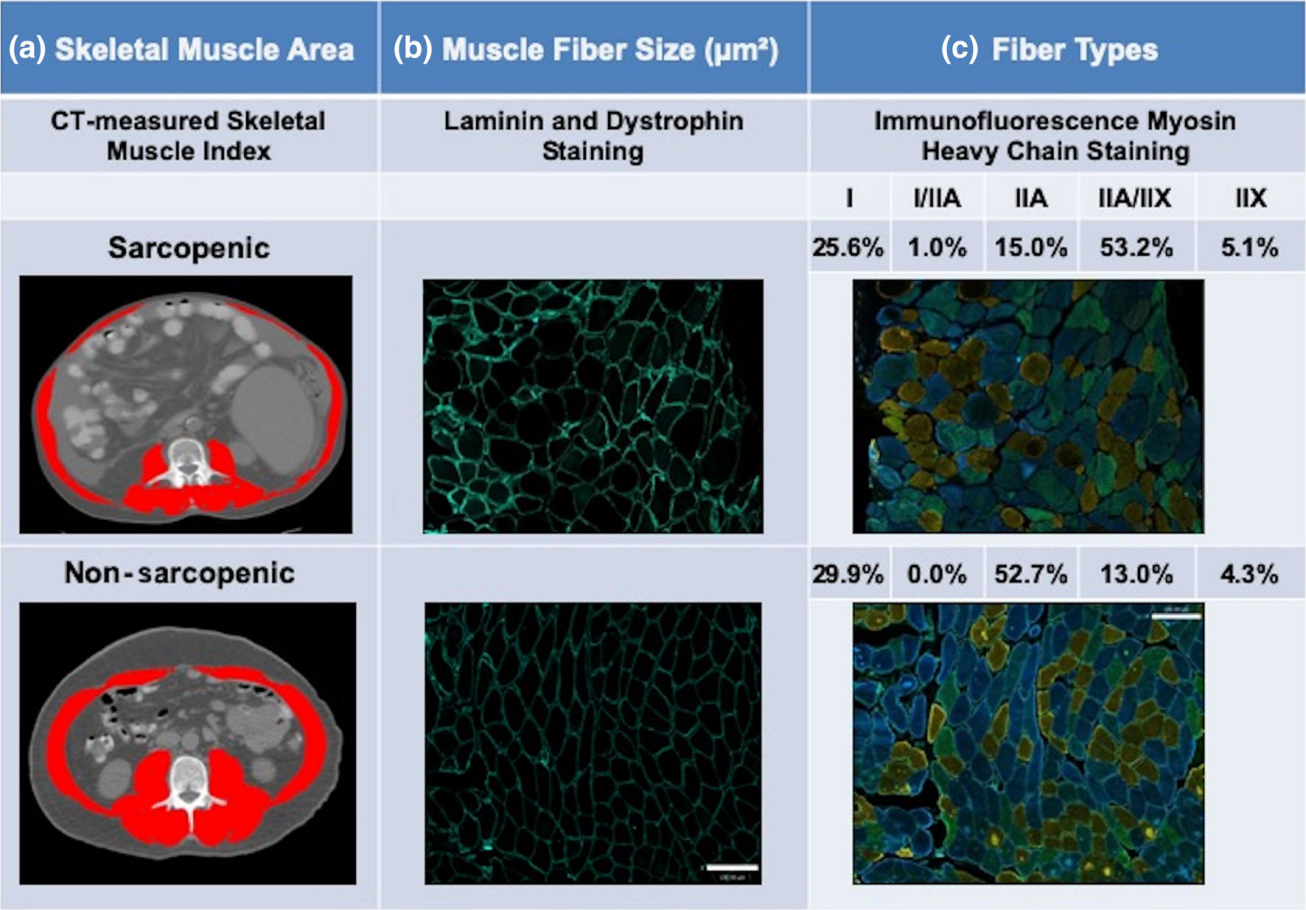
Abdominal computed tomography images taken at the 3rd lumbar vertebra to quantify total muscle cross-sectional area and rectus abdominis muscle morphological characteristics. Reduction in skeletal muscle mass in sarcopenia, identified by CT images, is detectable at the microscopic level by reduction in muscle fiber size and muscle fiber type transformation. **a** Red color designates skeletal muscles. Sarcopenic patient had had low SMI (43 cm^2^/m^2^) whereas in non-sarcopenic patient, SMI was 51 cm^2^/m^2^. **b** Muscle fiber boundaries were demarked using laminin and dystrophin stain (cell membrane) for muscle fiber size calculation. **c** Fiber types were identified in rectus abdominis muscle by using immunofluorescence myosin heavy-chain staining technique. Sarcopenic patient has mostly type IIA/IIX fibers (blue/green) whereas type IIA fibers (blue) were more frequent in non-sarcopenic patient. Scale bar = 130 µm

We recently investigated fiber-type atrophy in patients with cirrhosis using rectus abdominis (a muscle group identified at L3 CT image and accessible during LT) biopsies. Preliminary results from our research group suggest that reduction in skeletal muscle mass in sarcopenia identified by CT images is detectable at the microscopic level by reduction in muscle fiber size. Muscle fiber type transformation was also observed in a sarcopenic patient when compared to the non-sarcopenic patient (Fig. 3). Reduced skeletal muscle fiber size (median of 1552 versus 2475 µm^2^) and shift toward wider fast-twitch fibers (increased proportion of type IIA/IIX fibers concurrent with lower proportions of type IIA fibers) were visible in the muscle of a sarcopenic patient. The influence of sarcopenia in cirrhosis on both muscle fiber size and type needs to be investigated in larger prospective studies.

## Interventions to reverse sarcopenia

Muscle loss in cirrhosis is multifactorial and a combination of nutritional, physical, and pharmacological interventions might be necessary in reversing sarcopenia in cirrhosis. Although reduced food intake alone does not explain muscle loss in cirrhosis, high energy and protein diet may help to retain nitrogen balance [92]. Recent European Association for the Study of the Liver (EASL) Clinical Practice Guidelines on nutrition in chronic liver disease recommend protein intake of 1.2–1.5 g/kg/day and energy intake of at least 35 kcal/kg in non-obese patients (BMI < 30 kg/m^2^) [93]. Moreover, fasting longer than 6 h should be avoided by taking small, frequent meals, especially a late-night snack containing 50 gr complex carbohydrate [94] or BCAA to decrease lipid oxidation and improve nitrogen balance [95].

Vegetable proteins, compared to meat-based proteins, are poor in sulfated amino acids methionine and cysteine. Modifying the nitrogen source might be favorable in patients with chronic HE to improve their mental status without further loss of lean body mass. Vegetable proteins, depending on the food source, might contain the same nutritional value as animal protein. Legumes, for example, are high in leucine which can stimulate protein synthesis [96].

Nutritional deficits, such as low serum levels of BCAA, are frequent in patients with cirrhosis. Lower levels of BCAA in plasma and muscle have been prompted by hyperammonemia [97]. Other factors such as starvation, hyperinsulinemia and elevated BCAA muscle uptake have been linked to the pathogenesis of BCAA deficiency in patients with cirrhosis [51]. BCAAs contribute to both energy metabolism and protein synthesis in muscle. As an energy substrate, they are deaminated to provide carbon skeleton for TCA cycle. Complete oxidation of BCAAs leads to ammonia and glutamine production in muscle. BCAA supplementation (0.45 g/kg body weight; 45.5% leucine, 30% isoleucine, and 24.5% valine) as a single dose enhanced BCAAs uptake, glutamine production and ammonia metabolism in muscle of patients with cirrhosis, but also led to a substantial rise in blood ammonia levels. Significant glutamine release from the muscle and secondary deamidation of these newly released glutamine in other organs such as the small bowel and the kidney may contribute to this elevation [98]. Contrary to a previous study investigating the immediate effect of a single-dose BCAAs, long-term therapeutic trials reported reduction in blood ammonia levels following BCAA supplementation (12 g/day for 2 years) [99]. Therefore, prospective trials should investigate the beneficial impact of BCAAs on muscle and ammonia metabolism considering the dose, composition, and duration of BCAA supplementation. BCAA supplementation (952 mg l-isoleucine, 1904 mg l-leucine, and 1144 mg l-valine.), three times daily after meals for 48 weeks, improved muscle glucose uptake, muscle mass, and survival in patients with cirrhosis, which was concurrent with elevation in serum albumin levels [100]. Patient total energy and protein intake were adjusted to 25–35 kcal/kg/day and 1.0–1.4 kg/day, respectively, in accordance with European Society for Parenteral and Enteral Nutrition guidelines [101]. Contribution of the patient diet, in addition to the supplement intake, to the particular nutrient of interest needs to be considered in nutrient interventional studies.

Among BCAAs, leucine has demonstrated significant benefit on mTOR signaling [102]. Enhanced metabolic need for leucine in cirrhosis might be related to its increased mitochondrial oxidation to generate acetyl-CoA in hyperammonemic states [55]. Moreover, beneficial effects on sarcopenia have been found with leucine and its active metabolite, β-hydroxy-β-methylbutyrate (HMB) [103]. Most of the HMB is synthesized from leucine in the liver [104]. Considering hepatic failure and elevated leucine oxidation in skeletal muscle, diminished leucine plasma levels and, subsequently, impaired HMB synthesis occur in cirrhosis [51]. HMB exerts anti-catabolic effects in skeletal muscle and is suggested as a nutritional supplement [104].

Leucine-enriched BCAA supplementation (7.5 g l-leucine, 3.75 g l-isoleucine, and 3.75 g l-valine) at a single dose reversed disrupted mTOR1 signaling and decreased autophagy in skeletal muscle of patients with alcoholic cirrhosis. No changes in myostatin expression and ubiquitin–proteasome degradation were observed. Supplementation in this study was associated with reduction in other essential amino acid plasma levels suggesting that other essential amino acids should be included in supplements to stimulate muscle protein synthesis [105]. Increased phosphorylation and activation of general control non-depressible 2 (GCN2), a sensor of intracellular amino acid deficiency and its target, eIF2, were reversed by l-leucine-enriched BCAA supplementation [55].

Oral leucine administration (10 g/day), combined with moderate physical activity for 12 weeks, improved tight muscle mass and quality of life in patients with cirrhosis [106]. The significance of exercise in reversing sarcopenia might be explained by stimulation of mTOR signaling, inhibition of muscle apoptosis by decreasing local TNF-α levels, stimulation of mitochondrial oxidative capacity and increased blood flow to the muscle [107].

A minimum of 30 min of moderate intensity exercise (combined aerobic and resistance in a 3:2 ratio) per day for 3–5 times per week is recommended. Resistance exercise might be more beneficial in sarcopenia prevention and treatment [108].

Ammonia-lowering therapy with a combination of rifaximin and l-ornithine l-aspartate lowered plasma and muscle ammonia concentrations and improved muscle mass in an experimental model of hyperammonemia. Decrease in gastrocnemius muscle fiber size was partially reversed with a significant increase in the type II fibers. Ammonia-lowering therapy decreased expression of myostatin, autophagy markers and reversed GCN2/eIF2α phosphorylation as well as mTORC1 signaling which were altered by skeletal muscle hyperammonemia [109].

Carnitine plays an important role in fatty acid oxidation and around 25% of it is produced by the kidney and liver. l-Carnitine (1000 mg/day) administration for more than 6 months suppressed skeletal muscle loss in patients with cirrhosis. Ability of l-carnitine to reduce ammonia levels and improve mitochondrial function may contribute to prevention of skeletal muscle mass loss in patients with cirrhosis [110]. However, recent studies found that suppression of sarcopenia progression by L-carnitine in cirrhosis seems to be dose dependent and administration of high-dose L-carnitine (≥ 1274 mg/day) was associated with reduction in serum ammonia levels at a year following administration [111].

The ability of anabolic steroid, testosterone, to improve muscle mass in cirrhosis was investigated in previous studies. Lower testosterone levels were detected in sarcopenic male patients with cirrhosis compared to non-sarcopenic patients [112]. A 1-year double-blind, placebo-controlled trial of intramuscular testosterone administration to men with cirrhosis and low serum testosterone levels led to substantial development of muscle mass [113]. Anabolic impact of testosterone on muscle might be related to the suppression of muscle cell apoptosis and myostatin production [114].

Reversal of muscle loss or regain of muscle mass is the goal of therapeutic interventions with anti-inflammatory agents. Long-chain n-3 fatty acids have demonstrated capacity to increase skeletal muscle fatty acid oxidation and improve insulin sensitivity. However, anabolic stimulus, such as amino acids are required for mTOR activation by n-3 fatty acids and increase in muscle protein synthesis [115]. Peri-operative immunonutrition enriched in n-3 fatty acids (2 g), arginine (7.5 g), and nucleotides (0.8 g) for the median of 56 days in patients undergoing LT, had no significant impact on the total body protein and nutritional status as assessed by mid-arm muscle circumference [116]. However, the duration of supplementation (0–480 days) varied significantly between patients, which might contribute to the discrepant results.

Vitamin D supplementation improved sarcopenia in geriatric population. This might be related to the regulation of myoblast proliferation and differentiation through vitamin D receptors which are expressed in muscle fibers [117]. Although vitamin D deficiency has been linked to mortality in patients with chronic liver diseases [118, 119], its association with sarcopenia prevention or treatment in cirrhosis has not been investigated.

Earlier intervention at a time when anabolic potential exists, might be more effective than intervention at a refractory stage of muscle wasting. In an oncologic population, anabolic potential exists further away from death and suggests that interventions aimed at attenuating muscle wasting should be implemented earlier in the disease trajectory [120]. Further trials aiming to identify the appropriate time to initiate interventions to preserve muscle tissue and attenuate muscle wasting in patients with cirrhosis are required.

We acknowledge that the majority of these interventional studies included small number of patients with short-term intervention/follow-up time, but these studies raise additional, important question to be addressed in future studies. For instance, various factors such as dose, type, and duration of supplementations, etiology of cirrhosis, amount of dietary protein intake, time point of the diseases trajectory, compliance to the supplementation, and amount of physical activity with and without supplementation should be the focus of future large randomized controlled trials investigating both prevention and treatment of sarcopenia in patients with cirrhosis.

## Conclusion

Numerous factors such as malnutrition, diminished hepatic glycogen synthesis, increased muscle protein degradation, impaired mitochondrial function, and portal hypertension complications contribute to sarcopenia in cirrhosis. Multiple intracellular signaling pathways such as the IGF1/AKT, myostatin, cytokine/NFκB, and AMPK pathways interact and regulate muscle mass and protein metabolism, which are disrupted in cirrhosis. Increased muscle proteolysis might play an important role in the initial stages of muscle loss; however, at advanced stages, impaired satellite cell function and lower protein synthesis dominate.

Besides energy and protein intake in accordance with clinical guidelines, interventions with BCAA, testosterone, carnitine, ammonia-lowering therapies in combination with moderate exercise have the potential to reverse sarcopenia in cirrhosis. Cross-sectional imaging has enabled the capability to assess the efficacy of interventional approaches to reverse sarcopenia. Therefore, the efficacy of pharmacological and non-pharmacological interventions in reversing sarcopenia should be investigated using sensitive measures of muscle mass in future controlled clinical trials.
